# Oxidative stress impacts carbon metabolism and induces upregulation of redox enzymes and small secreted proteins in the wood-decay fungus *Phlebia radiata*

**DOI:** 10.1186/s40694-026-00222-2

**Published:** 2026-09-11

**Authors:** Janina Österman-Udd, Eero A. Kiviniemi, Asko Simojoki, Matti Wahlsten, Taina Lundell

**Affiliations:** 1 Department of Microbiology, Faculty of Agriculture and Forestry, University of Helsinki, Helsinki, Finland; 2 Department of Agricultural Sciences, Faculty of Agriculture and Forestry, University of Helsinki, Helsinki, Finland

**Keywords:** White rot fungus, Wood decay, Basidiomycota, Oxidative shock, Stress response, Gene regulation, Differential gene expression, Metabolic pathways, Pentose catabolic pathway, Small secreted proteins

## Abstract

**Background:**

Fungi live in diverse environments requiring tolerance against abiotic and biotic stress and changing atmospheric conditions. Studies with white rot and brown rot species of Polyporales Basidiomycota have demonstrated that aerobic wood decay fungi may adapt to low oxygen and even anoxic conditions, which they undoubtedly encounter in their deadwood habitat. In the white rot fungus *Phlebia radiata*, oxygen depletion on lignocellulose substrates leads to hypoxia and fermentative metabolism. In this study, we elaborated the atmospheric effect further by subjecting the fungus to oxidative stress on wood substrate under aerobic and low oxygen conditions, with the aim to examine changes in gene expression and metabolic pathways as consequences of the oxidative treatment.

**Results:**

Overall, 762 genes were significantly differentially expressed (DEGs with absolute Log_2_FoldChange > 1) 18 h after treatment with hydrogen peroxide according to RNA-Seq data. Half of these (348 genes) were downregulated in low oxygen (< 10% O_2_) cultures, with 185 genes unique to the condition but one third (121 genes) of unknown function. In aerobic cultures, a different response was observed with less DEGs showing downregulation (236 genes) while a higher number were upregulated (267 genes) and 153 of the upregulated genes were unique to the condition. Among DEGs upregulated under both conditions, small secreted proteins (SSPs) were the most abundant, followed by short-chain dehydrogenase/reductases, aldo-keto reductases, GNAT family acetyltransferases and CAZy carbohydrate active enzymes. Among downregulated DEGs, numerous SSP and CAZy classes, and genes encoding heat-shock proteins and MFS transporters were identified. Closer examination of carbon metabolism genes showed that under both conditions, oxidative treatment led to suppression of pentose catabolic pathway, glycerol metabolism, and formation of ethanol and acetate.

**Conclusions:**

Oxidative stress caused a substantial change in fungal gene expression, but with different responses depending on the culture atmosphere. This may be explained by contrasting fungal metabolic states before the shock as was observed in extracellular enzyme, aromatic metabolite and redox activity profiles. Surprisingly, oxidative shock caused downregulation of a few heat-shock proteins whereas small secreted proteins were either up- or downregulated, suggesting both sensing and regulative roles for these, functionally yet unknown, diverse fungal proteins.

**Supplementary Information:**

The online version contains supplementary material available at https://doi.org/10.1186/s40694-026-00222-2.

## Background

 Oxidative stress is one the most common environmental effects on fungi encountered irrespective of their lifestyle and ecological niche. Adaptive response to oxidative stress is a fundamental mechanism for survival in fungi including complete cellular “reprogramming” at gene transcriptional, RNA post-transcriptional, and metabolomic levels [1]. Among saprotrophic fungi like the wood-decaying Basidiomycota, however, little is known about stress-responsive mechanisms despite extensive genomic studies demonstrating conservation of sets of genes functioning in mitigation of oxidative conditions [2]. Previous studies demonstrate that atmospheric oxygen levels support fungal growth and wood degradation irrespective of the decay type [3–6]. More recently, investigations on fungal metabolic or genetic response to specific atmospheric effects like anoxia [7, 8], low-oxygen - elevated carbon dioxide fermentative conditions [9–11], high-oxygen toxicity [12] and potential oxidative damage [13] have, however, widened the view of wood-decay fungal physiology and adaptability under the changing environmental conditions.

Wood decay fungi generate and utilize hydrogen peroxide and other ROS (reactive oxygen species) in their extracellular biodegradative reactions and in responses that are similar to oxidative activities encountered with plant and human pathogenic fungi [2, 14]. Hydrogen peroxide that is generated intracellularly participates in cellular metabolism and acts as a redox signalling molecule [15]. In excess, extracellular H_2_O_2_ may cause abiotic shock leading to oxidative stress responses. Such stress, in connection to compromised antioxidant systems, may lead to cellular death [1]. Adaptive responses to oxidative stress is a fundamental mechanism for survival in fungi, including overall cellular responses at gene transcriptional, post-transcriptional, and metabolomic levels [1].

Together with effective intracellular enzyme mechanisms for tolerance against oxidative stress and accumulation of ROS, filamentous fungi of Basidiomycota produce sets of secreted oxidoreductases for production or quenching of extracellular H_2_O_2_ [2, 14, 16]. Especially the secreted class II peroxidases (carbohydrate active enzyme (CAZy) AA2 family manganese, versatile and lignin peroxidases), dioxygen-consuming laccases and other oxidases, and the lytic polysaccharide mono-oxygenases (LPMOs) are essential enzymes in degradation of wood components. Although these enzymes tolerate oxidative conditions, ROS and reactive radicals, exogenously added high dosages of hydrogen peroxide may cause transient inhibition or even inactivation of their catalytic activities [13, 17, 18].

*Phlebia radiata* is a white rot fungal species of Basidiomycota (class Agaricomycetes, order Polyporales, family Meruliaceae) efficient in wood decay, including decomposition of lignin [19–21]. The fungus has been applied in bioconversions of wastes and wood into bioethanol [10], production of natural compounds [22] as well as in degradation of toxic xenobiotics, dyes and aromatic compounds [19, 23, 24]. Interactions of the species with other fungi and microbes are important in natural wood decay in Nordic forest ecosystems, more recently also being investigated under controlled co-cultivation conditions on wood and lignocellulose substrates [25–27].

Genome sequencing of the isolate 79 (FBCC 0043) showed a typical Polyporales fungal genome structure with a wide repertoire of genes encoding CAZymes (carbohydrate active enzymes), multiple families of auxiliary oxidoreductases, and biosynthetic genes for various classes of secondary metabolites, especially terpenoids [28]. Due to the ability to secrete sets of extracellular hydrolytic CAZymes and oxidoreductases of laccase and class-II peroxidase families [20, 21, 29], we investigated the proteome and transcriptome of the fungus on wood substrate [11, 30]. The switch to fermentative metabolism and ethanol production upon wood degradation under oxygen depletion (high CO_2_, low O_2_) revealed several potential transcription factors involved, and upregulation of the fungal phosphoketolase pathway [11].

With all this in mind, we previously observed the effect of oxidative stress on a wood decay fungus first at overall growth, enzyme activity and natural product production level, on wood substrate but under differing atmospheric conditions [17]. In the current research, we aimed to explore how the atmospheric conditions affect fungal metabolism at gene expression level, looking to answer (i) whether there are there general or more specific transcriptomic changes caused by the severe oxidative treatment under these conditions, and (ii) whether the fungus has an ability to recover from the oxidative shock within a few days. Our analysis of differential gene expression shows a substantial change in fungal gene expression after the oxidative shock, but with slightly different responses dependent on the culture atmosphere. These similarities and differences at gene expression and metabolic pathway level are presented and discussed.

## Materials and methods

### Fungal isolate

*Phlebia radiata* 79 (FBCC 0043) is a genome-sequenced and phylogenetically confirmed [21, 25, 28] original wild type isolate from Finland stored in the FBCC subcollection of Microbial Domain Biological Resource Centre HAMBI (https://www.helsinki.fi/en/infrastructures/biodiversity-collections/infrastructures/microbial-domain-biological-resource-centre-hambi), University of Helsinki. The fungus was maintained as hyphal culture on 2% (w/v) malt extract (Neogen) 2% (w/v) agar (MEA) plates at room temperature in the dark.

### Cultivation on birch wood sawdust

The fungus was cultivated on silver birch (*Betula pendula*) sapwood sawdust (particle size below 4.8 mm) as static (non-agitated) semisolid laboratory cultures. Before inoculation, 2 g of oven-dried sieved sawdust was dry-autoclaved (121 °C, 15 min) in 100 ml conical glass flasks, and 18 ml of autoclave-sterilized 0.1% (w/v) yeast extract (Neogen) water solution was added into each flask. Fungal cultures were initiated with 2 pieces of 6 mm diameter mycelial plugs cut from seven-day grown MEA plates and inserted on top of the wetted sawdust. Culture flasks were closed with sterile cellulose stoppers and aluminium caps, and incubated at 25 °C in the dark. On growth day 5, autoclave-sterilized tight rubber plugs with multiple inlet valves [10, 17] were installed onto half of the culture flasks (low oxygen cultures, LO) while cellulose stoppers were left in place for the rest of the flasks (aerobic cultures, AE). For both atmospheric conditions, there were 5 parallel culture flasks (biological replicates) for each sampling timepoint. A set of no-fungus (NF) control flasks containing uninoculated sterile pieces of MEA was prepared in a similar way as the LO cultures, with four parallel flasks for each sampling timepoint.

### Oxidative treatment and sample collection

On cultivation day 10, oxidative shock treatment was generated by addition of hydrogen peroxide (H_2_O_2,_ Fischer Scientific) into half of the flasks (10 fungal culture flasks per atmospheric condition) to a final concentration of 0.1% (w/v) (35 mM). The addition was done through the plug valves for the LO cultures (both fungal cultures and no-fungus controls) using the same strategy as previously reported [17]. One set of five culture flasks for both atmospheres were left untreated by adding only sterile ultrapure water instead. Before the treatment (on cultivation day 10), and 18 h (on day 11) and four days (on day 14) after exposure to H_2_O_2_, five parallel culture flasks for each treatment and culture atmosphere were completely harvested by separating the solids (wood substrate and mycelium) from culture supernatant by filtering through clean rayon-polyester fabric (Miracloth, Calbiochem, Merck Millipore). The solid substances were snap-frozen with liquid nitrogen and kept at -70 °C while the supernatants were separately stored at -20 °C for further analyses.

### Gas chromatography

On cultivation day 10 (before oxidative treatment) and on days 11 and 14 (18 h and four days after the treatment, respectively), gas samples were withdrawn from the headspace of the tightly plugged flasks (LO cultures) through the inlet valves into pre-evacuated gas chromatography vials using a helium-flushed syringe. Gas chromatography (GC) analysis of the headspace gas samples of the LO culture flasks was performed to determine consumption or evolution of O_2_, CO_2_, N_2_, CH_4_ and N_2_O as described previously [11, 31]. No-fungus LO control flasks were subjected to similar sampling and analysis to determine the effect of hydrogen peroxide treatment on the birch wood substrate yeast-extract medium.

### Fungal extracellular activities in the cultures

Enzyme activities in the culture supernatants were assayed as previously described for laccase, manganese peroxidase (MnP) and lignin peroxidase (LiP) [21, 22, 26, 32], xylanase and endoglucanase [33, 34] in 96-microwell plate scale using a multimode reader (Spark, Tecan, Switzerland).

Iron reduction capacity was analyzed in microplate scale adapting a previous method [35]. Briefly, 10 µl aliquot of the culture supernatant was mixed with 200 µl of 50 mM acetate buffer (pH 4.5) and 10 µl of 10 mM FeCl_3_ (freshly prepared in 50 mM acetate buffer, pH 4.5). The reaction was initiated by injecting 8 µl of 0.5% (w/v) ferrozine (Sigma-Aldrich; prepared in 50 mM acetate buffer, pH 4.5) into the reaction mixture, and measuring absorbance of the reaction product complex at 562 nm after five minutes of incubation in the dark at 25 °C. Absorbance change was compared against a standard curve of Fe^2+^ as FeSO_4_ at various concentrations (0.0 to 3.0 mM).

Aliquots (5 ml) of fungal (and NF control) culture supernatants were lyophilized, then solubilized in 1 ml of 70% (v/v) methanol, and stored at -20 °C for further analyses. Antimicrobial assay and DPPH antioxidant activity were determined for these extracts similarly as described previously [17]. Additionally, these extracts were analyzed using UPLC-PDA-MS for identification and quantification of fungal-generated veratryl compounds and wood substrate-derived compounds [22].

### RNA extraction

Frozen (at -70 °C) solid substances (substrate wood and mycelium) from three replicate culture flasks for each treatment and timepoint were selected for RNA-extraction. Samples were kept frozen on dry ice and ground into powder under liquid nitrogen using an analytical mill (A11 basic, IKA, Germany). Glassware and metal items were heat-sterilized, while all other devices and equipment were cleaned with RNaseZap (Thermo Fisher Scientific) and diethyl pyrocarbonate (DEPC)-treated ultrapure sterile water before use. RNA was extracted from the powdery matter using guanidine thiocyanate buffer – CsCl gradient centrifugation method [11, 30, 36], solubilized in 100 ml of DEPC-treated water and purified with NucleoSpin RNA Clean-up kit (Macherey-Nagel) according to manufacturer’s instructions. RNA concentration was measured with NanoDrop One (Thermo Fisher Scientific). Purified total RNA samples were kept at -70 °C before being sent to sequencing.

### RNA sequencing and treatment of sequence reads

RNA-Seq reads were generated from total RNA by BGI Genomics (Shenzhen, People’s Republic of China). mRNA enrichment by oligo dT selection was followed by cDNA generation using random primers. Eukaryotic Strand-specific mRNA libraries were sequenced as PE100 sequencing on the DNBSEQ platform, generating about 20–25 M read pairs per sample. RNA extraction and sequencing was done in four batches (batch 1: aerobic cultures on day 10 and 11, AE10 and AE11; batch 2: low oxygen cultures on day 10 and 11, LO10 and LO11; batch 3: aerobic cultures on day 14, AE14; batch 4: low oxygen cultures on day 14, LO14).

The obtained “clean reads” were quality-checked using FastQC version 0.11.8 [37]. A subset of 200,000 read pairs were sampled with seqtk version 1.3-r106 [38] and aligned to the *P. radiata* genome [28] with STAR aligner version 2.7.8a [39]. These alignments were analysed with the RSeQC v3.0.1 [40] module infer_experiment.py to confirm the strandedness, and indexed using samtools 1.10 [41, 42] to enable visual inspection in Tablet v1.21.02.08 [43]. All reads were subsequently aligned to the genome using STAR. Alignment metrics were obtained with Picard Tools 2.21.4-SNAPSHOT [44] tool CollectInsertSizeMetrics, bamtools v2.5.1 stats command [45], as well as RSeQC module bam_stat.py. Bowtie2 v2.4.1 [46] was used to align all reads to the rDNA gene region of genomic scaffold_4, in order to analyse the abundance of reads originating from rRNAs.

### Differential gene expression analysis

After alignment and generation of gene counts, the columns corresponding to stranded reverse data counts from every sample were joined into one big count table. This count table was provided as input, together with a phenodata table, to differential expression (DE) analysis performed in R version 4.0.5 (2021-03-31) -- “Shake and Throw” [47] using the DESeq2 package [48]. Prior to DE analysis, rows containing genes with zero count in all samples were removed from the count table. A principal component analysis (PCA) was performed based on raw counts transformed with the variance stabilizing transformation to visualize the samples, with colours from the RColorBrewer package [49]. Statistics for differential expression were generated from a DESeqDataSet (with design ~ batch.group) using FDR threshold 0.05 and log_2_FoldChange threshold 1.0 (alpha = 0.05 and lfcThreshold = 1.0 in the results() function of DESeq2), and the different comparisons were done using contrasts.

### Metabolic pathway and protein cellular function predictions

Some gene models previously predicted for *P. radiata* [28] were revised for the bioinformatic analyses performed in this study. The genome sequence with updated gene models and functional annotations was submitted to the European Nucleotide Archive (ENA) (GCA_979071105). The updated gene models were further annotated with KEGG orthology (KO) identifiers using KofamScan (v1.3.0 [50]). Lists of all KO terms and pathways were downloaded via the KEGG API [51]. Pathways irrelevant for fungi were excluded, and K-numbers assigned to *P. radiata* genes mapped to KEGG pathways. KEGG enrichment analyses were performed in R using libraries tidyverse [52], clusterProfiler (v4.10.1 [53], , using ‘fgsea’ for GSEA analysis [54]) and enrichplot (v1.22.0 [55]), . For GSEA, genes were ordered based on shrunken LFC values generated with the apeglm shrinkage estimator [56]). The clusterProfiler ‘GSEA’ function was run with default settings (including a pvalueCutoff of 0.05), except for the minimal size of each geneSet which was set to 5. Our customized mapping data was used as input to TERM2GENE and TERM2NAME. The clusteProfiler package function ‘enricher’ was also used for over-representation analysis (ORA) of functional categories of genes in the sharedUp and sharedDown gene sets identified from the DE analysis. For this analysis, the functional annotations were used as such, but all identified genes of the categories SSPs, CAZys, HSPs, SDRs, MDRs, MFS transporters, Transcription factors, Old Yellow Enzymes, Terpene cyclases, NTPase family proteins, and GNAT family acetyltransferases were grouped under these custom terms. The background gene set provided to the universe parameter was the set of genes detected in the RNA-Seq samples where the sum of raw counts over all 24 samples was > 30 (12133 genes), and a minGSSize of 5 was used for analysis. For genes that were more closely inspected based on DE analysis results, functional annotations were generally verified by blastp searches against the NCBI nr database as well as searches for conserved protein domains via CDD-Search [57–59].

### Data visualisation

OriginPro software [60] was used for graphical presentation of numeric data obtained for enzyme and biological activities, and headspace gas content in the fungal cultures. For visualisation of the transcriptomic data, log_2_FoldChange values of DEGs defined in the day11 vs. day10 comparison were visualized with heatmaps, using the functional categories defined for the ORA with at least 3 DEGs in the category. These figures were generated with R version4.5.3 (2026-03-11 ucrt) -- “Reassured Reassurer”, using packages tidyverse [52] (version 2.0.0, including ggplot2 version4.0.3, dplyr version 1.2.1 and stringr version 1.6.0),, circlize [61] (version 0.4.18) and ComplexHeatmap [62] (version 2.26.1).

## Results

### Effect of oxidative shock on enzyme activities and culture atmosphere

**Fig. 1:**
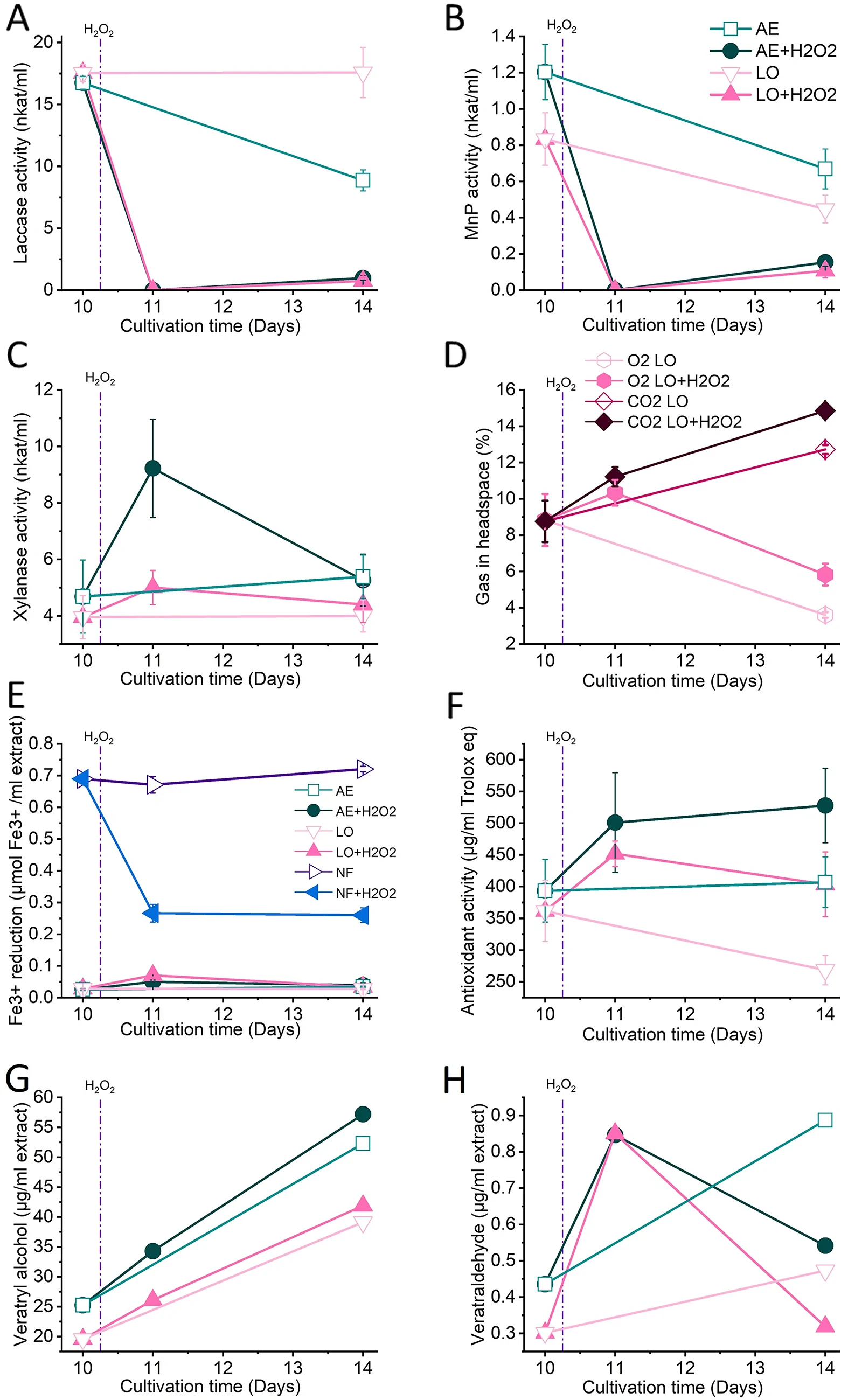
Extracellular enzyme and antioxidant activities, production of veratryl compounds, and changes in the headspace gas composition of fungal cultures maintained under two atmospheric conditions. Activities of **A** laccase, **B** manganese peroxidase, and **C** xylanase in culture supernatants; **D** relative abundance of headspace O_2_ and CO_2_ in low oxygen (LO) fungal cultures; **E** iron reduction, **F** antioxidant activity, **G** veratryl alcohol, **H** veratraldehyde in supernatant sample extracts. Dashed line: addition of H_2_O_2_ into the cultures. *AE* aerobic fungal cultures, *LO* low oxygen fungal cultures, *NF* no fungus control flasks. For more details, see Materials and methods

To follow fungal vitality under the two atmospheric conditions before and after the treatment with hydrogen peroxide (on day 11 of the cultivation), specific extracellular redox enzyme (laccase and manganese peroxidase) activities and one CAZy hydrolytic (xylanase) activity were assayed. Gas samples were taken from the tightly closed low oxygen (LO) atmosphere cultures especially to follow the change in oxygen content and O_2_/CO_2_ ratio in the headspace after the treatment. Since the aerobic (AE) fungal cultures were closed with ventilating stoppers, they were assumed to maintain atmospheric 21% O_2_ conditions. In the low oxygen (LO) fungal cultures on birch wood, oxygen was depleted down to the level of 4% of the headspace atmosphere on cultivation day 14 (Fig. 1D). Addition of hydrogen peroxide caused a slight increase in the O_2_ level (from initial 8% to 10% on day 11), and this surplus could be observed until the end of the experiment. A similar difference was detected in the accumulation of CO_2_ (12% versus 14% on day 14) in these culture flasks. These headspace gas contents with lower O_2_/CO_2_ ratio indicate transition towards fungal fermentative metabolism (see Discussion; [11]).

Laccase and manganese peroxidase (MnP) enzyme activities in the fungal culture supernatants showed similar responses to the oxidative treatment performed by addition of H_2_O_2_ into the culture flasks (Fig. 1A, B). Enzyme activities of approximately 17 nkat/ml and 1 nkat/ml for laccase and MnP, respectively, as determined to be present in the fungal cultures on day 10, were fully abolished by the treatment (with no activities detected for either enzyme) after 18 h of incubation, on cultivation day 11. Within three days, some laccase (approximately 1 nkat/ml) and MnP (approximately 0.1 nkat/ml) activity could be detected again in the treated cultures (on cultivation day 14). However, only 1/20 to 1/10 activity levels were reached pointing to quick elimination of extracellular oxidoreductive activities (either by transient inhibition of enzyme catalysis or protein oxidation and denaturation) due to the oxidative treatment (Fig. 1A, B). In contrast, no such dramatic drop of activities was observed in the non-treated fungal cultures, and on day 14 both laccase and MnP activities remained at high levels (about ½ of day 10 activity values). Gene expression data, however, indicated inconsistent expression patterns for distinct MnP (CAZy AA2) and laccase-encoding (CAZy AA1) genes (Fig. 3).

**Fig. 2:**
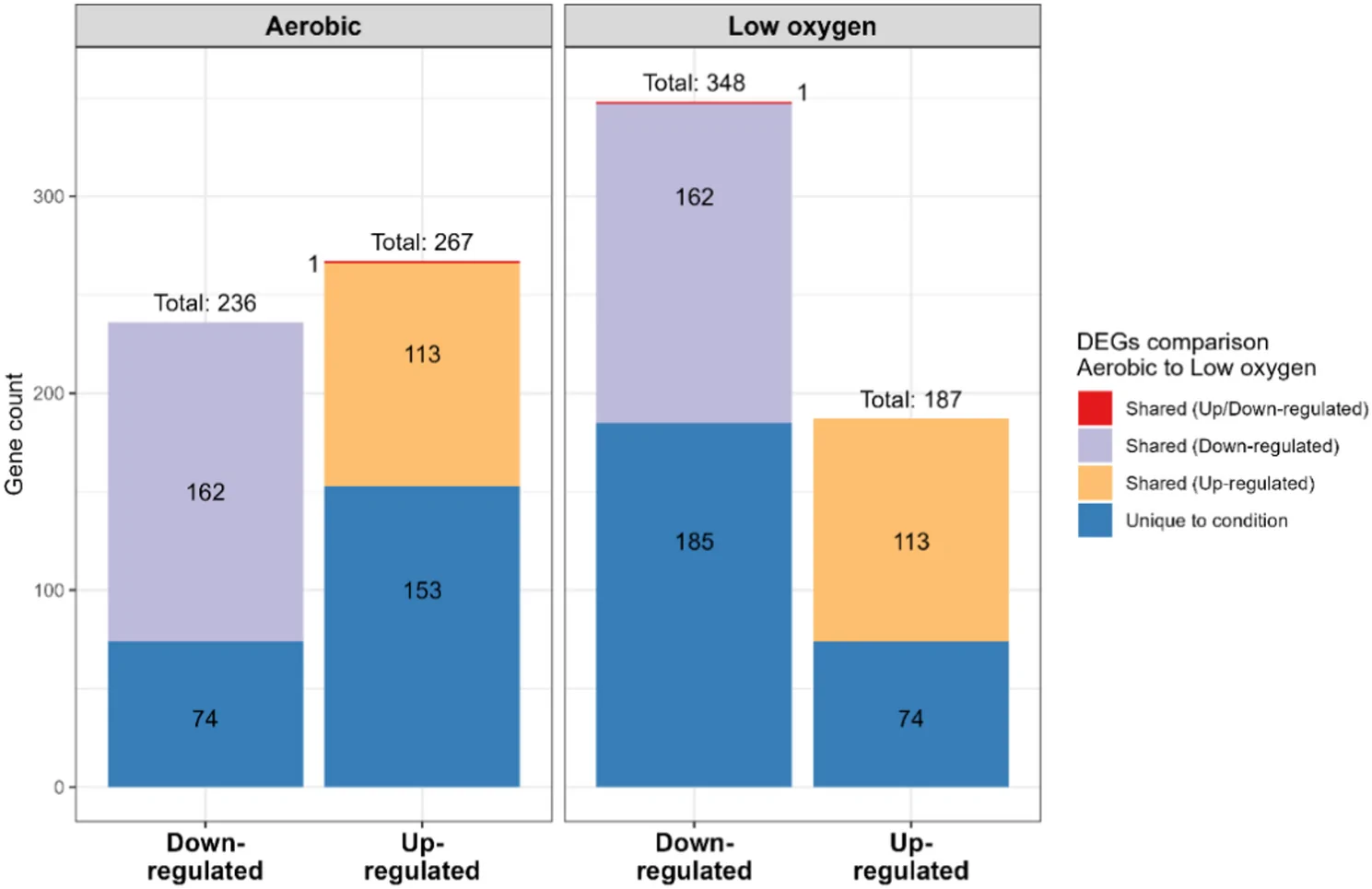
Differentially expressed genes (DEGs) detected in the fungal cultures 18 h after the oxidative shock. The numbers represent down- and up-regulated DEGs 18 h after exposure to H_2_O_2_ (on day 11) compared to gene expression before the treatment (on day 10) under aerobic and low oxygen cultivation conditions

A different response was observed, however, for xylanase activity which increased after the oxidative treatment, especially in the aerobic (AE) fungal cultures (Fig. 1C). Notable is the variation in xylanase activities between the five parallel cultures. On day 14, however, the treated and untreated fungal cultures returned to the previous range of activity (about 4–5 nkat/ml), indicative of a more quick and transient disturbance in xylanase enzyme presence and function. No lignin peroxidase or endoglucanase activity could be detected in any of the samples, most probably due to low initial activity and enzyme levels present in the fungal cultures before the treatment.

### Bioactivities and fungal compounds produced in the cultures

Antioxidant activities in the fungal cultures and birch-wood control incubations were followed by iron reduction and DPPH assays, to monitor potential changes caused by the oxidative treatment. As observed in our previous study under similar cultivation conditions [17], iron (Fe^3+^) reduction activities of the supernatant extracts were markedly lower in the fungal cultures than in the no-fungus (NF) wood substrate-medium control incubations (Fig. 1E). In fungal cultures, low but constant values were measured at the sampling timepoints regardless of the culture atmosphere and treatment. In contrast, H_2_O_2_ addition caused a marked decrease in iron reduction capacity in the NF control samples. This indicates elimination of wood and medium-derived chemical reductants by the oxidative treatment. On the contrary, DPPH antioxidant activities in the fungal culture extracts were slightly increasing after oxidative treatment both under AE and LO conditions (Fig. 1F), implying release of antioxidant compounds into the fungal cultures by H_2_O_2_ addition.

Veratryl (3,4-dimethoxybenzyl) alcohol and veratraldehyde (3,4-dimethoxybenzaldehyde) are well-characterised natural products of the fungus [22] and were detected in corresponding concentrations in the culture supernatants (Fig. 1G, H). Veratryl alcohol accumulated in the fungal cultures both under aerobic and low oxygen conditions, and H_2_O_2_treatment caused an increase in veratraldehyde concentrations. This increase was, however, transient whereas more steady accumulation of veratraldehyde was observed in the non-treated fungal cultures under both atmospheric conditions (Fig. 1G, H). Veratric acid was not detected as a product whereas a few other, yet unidentified, monomeric aromatic compounds were observed to accumulate in the supernatant extracts.

### General response of fungal gene expression to oxidative treatment

To attain an overview of the effect of the oxidative treatment on fungal gene expression, and to compare fungal genetic and metabolic responses under the two different cultivation atmospheres (aerobic AE, and low oxygen LO), mRNA was sequenced from 24 samples (representing three biological replicate cultures incubated under either AE or LO conditions, including H_2_O_2_-treated and non-treated fungal cultures harvested at three timepoints). The obtained RNA-Seq reads mapped to 12,450 *P. radiata* genes (out of the 14067 annotated genes in the GCA_979071105 genome assembly). A PCA of the raw data showed that the three biological replicates of each sampling set grouped together and were separated from the other samples of different atmospheric condition and sampling timepoint (Additional File 2). Analysis of differential expression demonstrated that, among the 12,450 genes expressed (defined as sum of reads assigned greater than zero), on day 11 (18 h after H_2_O_2_ treatment) altogether 762 genes were classified as significantly differentially expressed genes (DEGs) with a Log_2_FoldChange of at least 1, in comparison to cultivation day 10 (prior to oxidative treatment) when results from both atmospheric conditions (AE and LO) are combined (Fig. 2). Among these DEGs, there were 162 downregulated genes and 113 upregulated genes shared between both atmospheric conditions 18 h after the treatment (Fig. 2; Additional File 3).

**Fig. 3:**
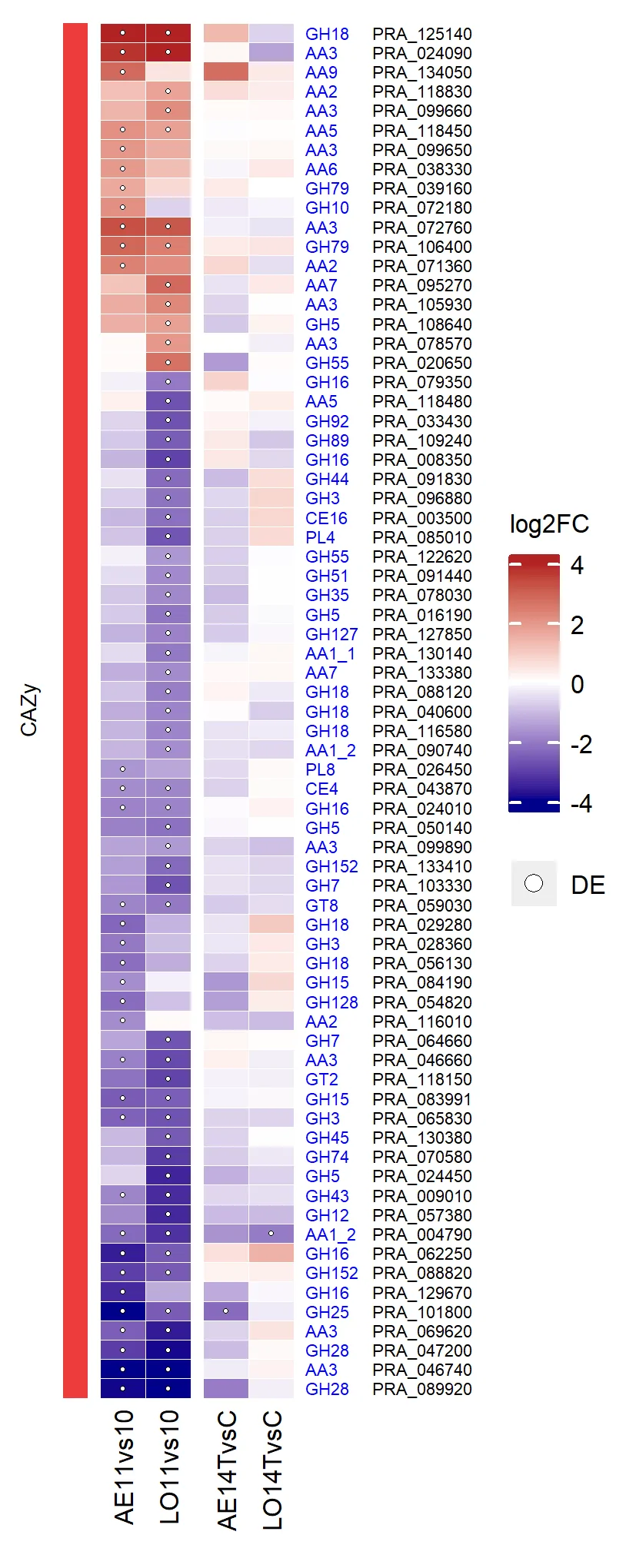
Log_2_FoldChange values of CAZy genes within the combined set of all DEGs. Log_2_FoldChange values are shown for aerobic (AE) and low oxygen (LO) fungal cultures on day 11 (18 h after exposure to H_2_O_2_) versus day 10 (before the treatment) and on day 14 (H_2_O_2_-treated sample 14T versus untreated sample 14 C). Differential expression is indicated with colouring (log_2_FC box). Gene identifiers with predicted protein CAZy classes (in blue) are indicated

In addition, there were genes differentially expressed only under either of the culture atmospheric conditions (unique up/down): 74 genes were downregulated in aerobic (AE) fungal cultures only whereas 185 genes were downregulated only in low oxygen (LO) cultures, while 153 and 74 genes were upregulated in AE cultures and LO cultures, respectively (Fig. 2; Additional File 3). In addition, only one of the DEGs shared between the two atmospheric conditions was found to be regulated in opposite direction (Fig. 2, shared Up-/Downregulated). In total, 503 and 535 genes were differentially expressed under AE and LO cultivation conditions, respectively, 18 h after exposure to H_2_O_2_.

**Fig. 4:**
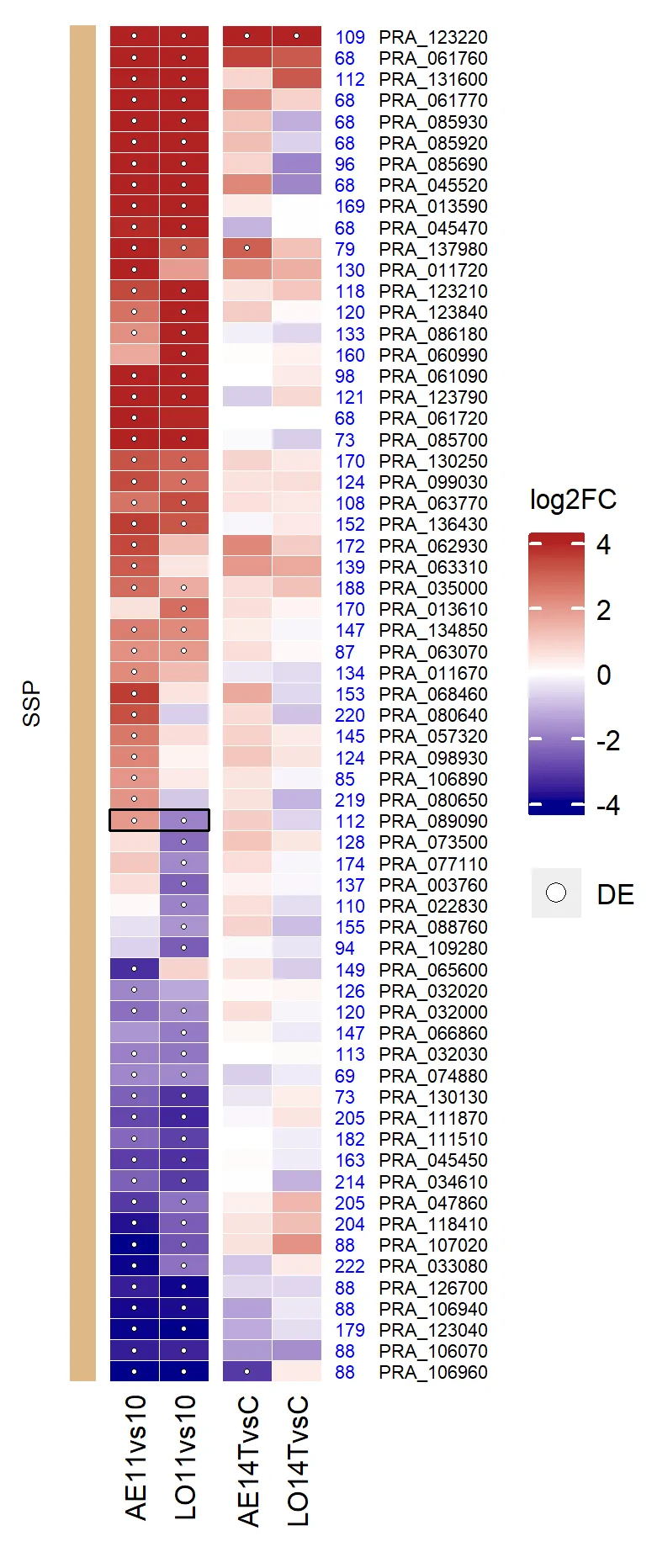
Log_2_FoldChange values of SSP genes within the combined set of all DEGs. Log_2_FoldChange values are shown for aerobic (AE) and low oxygen (LO) fungal cultures on day 11 (18 h after exposure to H_2_O_2_) versus day 10 (before the treatment) and on day 14 (H_2_O_2_-treated sample 14T versus untreated sample 14 C). Differential expression is indicated with colouring (log_2_FC box). For each gene identifier, predicted protein amino-acid length is indicated by blue numbers. The single gene significantly upregulated under AE atmosphere while significantly downregulated under LO is indicated with a black box

**Fig. 5:**
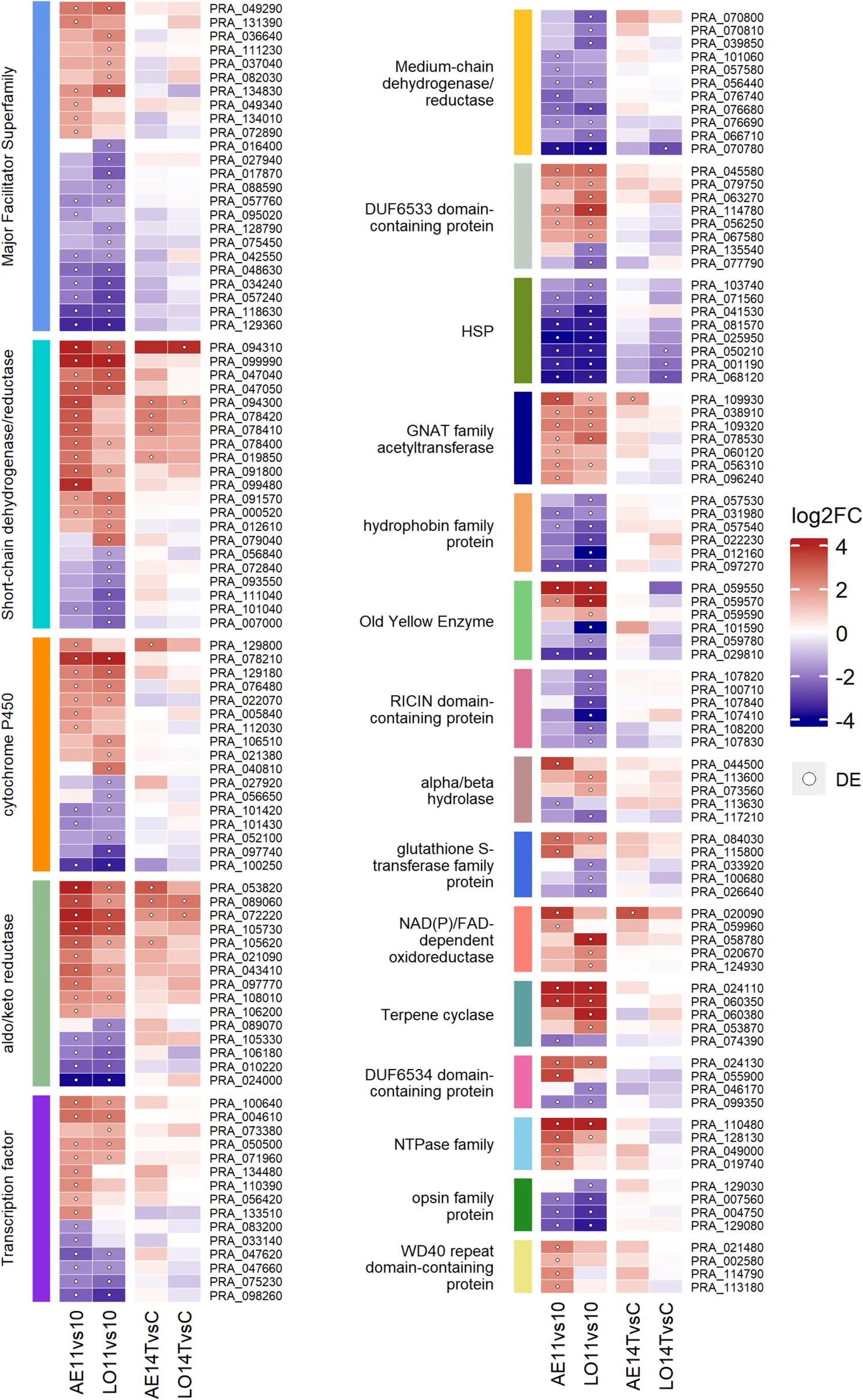
Log_2_FoldChange values of functional categories with at least 3 genes within the combined set of all DEGs excluding major categories of SSPs and CAZys (see Figs. 3 and 4). Log_2_FoldChange values are shown for aerobic (AE) and low oxygen (LO) fungal cultures on day 11 (18 h after exposure to H_2_O_2_) versus day 10 (before the treatment) and on day 14 (H_2_O_2_-treated sample 14T versus untreated sample 14 C). Differential expression is indicated with colouring (log_2_FC box)

In total, the oxidative treatment resulted in a slightly higher number of upregulated than downregulated DEGs found in fungal AE cultures, while there were relatively more downregulated DEGs observed under LO conditions (Fig. 2. When gene expression in H_2_O_2_-treated samples on day 14, that is four days after the oxidative treatment, was compared to the corresponding non-treated fungal cultures of the same timepoint, a total of 42 significantly differentially expressed genes was found (Additional File 3). Among these, however, 14 genes were not observed as DEGs in corresponding fungal cultures harvested directly after the oxidative treatment on day 11. Only 3 genes were differentially expressed both on day 11 and 14 under both atmospheric conditions (Figs. 4 and 5), while 20 DEGs were shared between day 11 and day 14 in AE cultures and 11 DEGs, correspondingly, in LO cultures (Additional File 3).

### Overall effect of the oxidative shock on cellular function and metabolic pathways

KEGG Orthology (KO) identifiers were assigned to the predicted protein sequences of *P. radiata* genes by HMMER/HMMSEARCH against KOfam (a customized HMM database of KEGG Orthologs) using KofamScan, resulting in 3932 protein-coding genes receiving a KO identifier (K number). The K numbers of 2273 genes could be mapped to KEGG pathways, and this pathway information was further used for enrichment analyses.

**Fig. 6:**
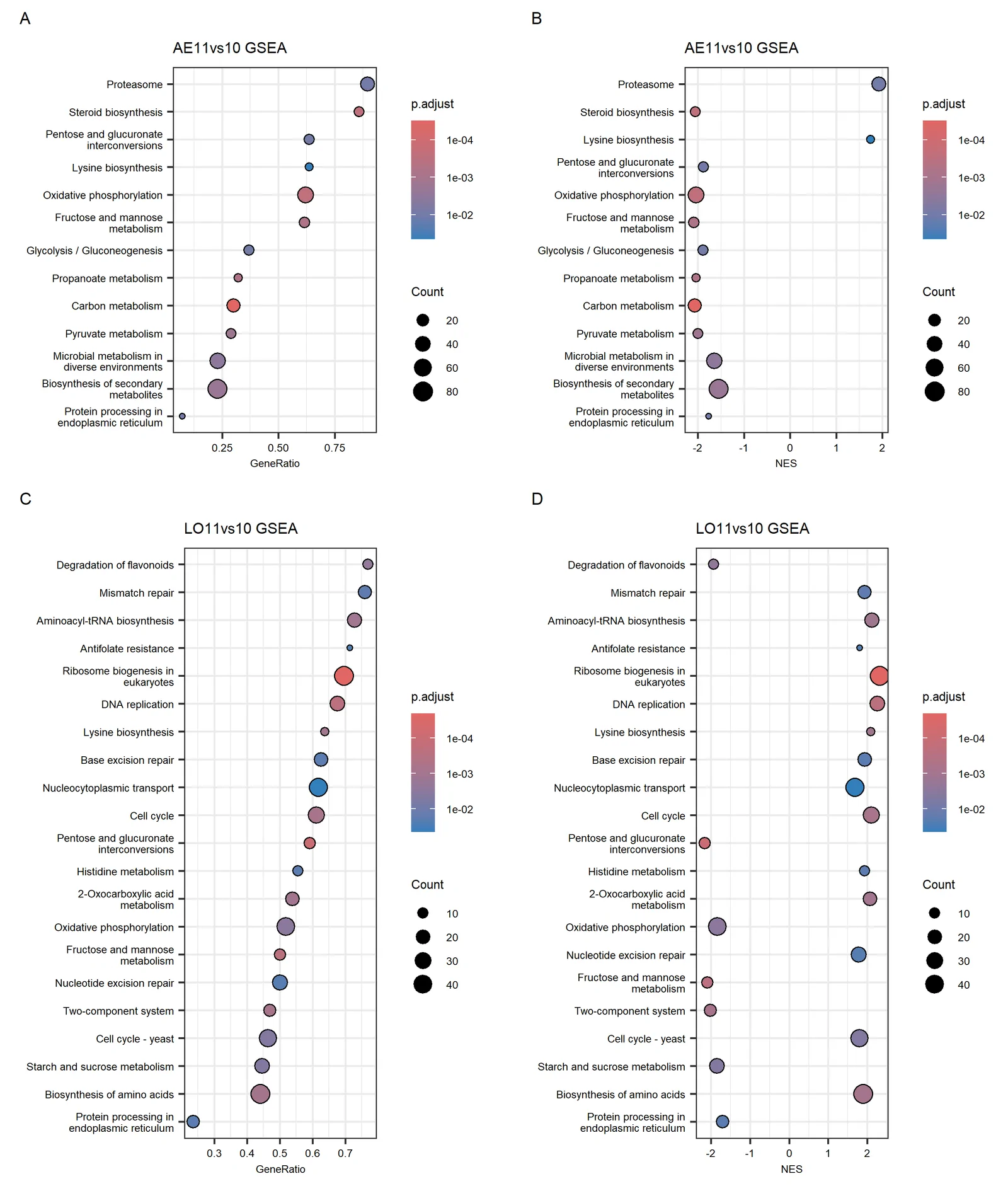
Enriched KEGG pathways among *Phlebia radiata* genes based on transciptome changes after H_2_O_2_ treatment. Analysis results are based on log_2_FoldChange values of (**A**, **B**) aerobic (AE) and (**C**, **D**) low oxygen (LO) fungal cultures on day 11 (18 h after exposure to H_2_O_2_) versus day 10 (before the treatment). In plots **A** and **C**, results are presented as dotplots based on GeneRatio, representing the ratio of core enrichment genes contributing most to the enrichment results compared to the total count of genes in the pathway for each enriched category. In plots **B** and **D**, the same results are presented based on normalized enrichment score (NES) values (enrichment score normalized to mean enrichment of random samples of the same size) reflecting up- and downregulation patterns of the enriched KEGG categories. Count as indicated by point size corresponds to core enrichment genes, and the p.adjust value is the enrichment p-value adjusted with the Benjamini-Hochberg method. Only enriched categories (with p.adjust < 0.05) are shown

Gene set enrichment analysis (GSEA) was performed on the full set of *P. radiata* genes to evaluate whether all fungal genes assigned to some specific KEGG pathway changed in a coordinated way as a response to oxidative treatment. Genes were ranked based on shrunken Log_2_FoldChange values assigned during the differential expression analysis. Gene ranks from analysis of the AE fungal samples from day 11 against day 10, and LO samples from the corresponding analysis, were included in GSEA in two separate runs. GSEA exposed distinct fungal gene expression patterns for the two different atmospheric cultivation conditions after exposure to H_2_O_2_ (Fig. 6).

Under aerobic (AE) conditions, the number of upregulated DEGs detected 18 h after the treatment was higher compared to the number of downregulated DEGs (Fig. 2). However, based on the GSEA considering all (KEGG pathway-mapped) genes, only proteasome- and lysine biosynthesis-related genes were generally upregulated (although only one (PRA_010870) of these genes was differentially expressed) (Fig. 6A, B). On the other hand, different metabolic pathways (Glycolysis/Gluconeogenesis, Pentose and glucuronate interconversions, Pyruvate metabolism, and especially Steroid biosynthesis, Carbon metabolism, Oxidative Phosphorylation, Fructose and mannose metabolism, and Propanoate metabolism) were generally downregulated based on this analysis (Fig. 6B). Under LO conditions, in contrast, a stronger effect on essential cell functions and repair mechanisms was observed (Fig. 6C, D).

Overall, the following KEGG pathways appeared to be generally downregulated after H_2_O_2_ exposure in both AE and LO fungal cultures: Protein processing in endoplasmic reticulum, Oxidative phosphorylation, Pentose and glucuronate interconversions, as well as Fructose and mannose metabolism (Fig. 6). This points to an overall shock effect and suppressive regulation of fungal primary metabolism and cell functions as an immediate response to oxidative treatment.

### Gene expression responses shared between aerobic AE and low oxygen LO conditions

The differentially expressed genes were subsequently inspected more closely to assess the oxidative shock effect on genes of different functional categories. To observe the general fungal response regardless of culture condition, the DEGs with analogous response to H_2_O_2_ treatment under both AE and LO culture conditions (day 11 samples compared to day 10 samples), hereafter called shared upregulated genes and shared downregulated genes (Fig. 2), were selected for further analysis (Table 1 Additional File 3). The DEGs identified were also ordered based on their Log_2_FoldChange values, and Top50 sets of most drastically up- and downregulated genes were inspected.

Regarding fungal gene expression under LO (low-oxygen) conditions, it is noteworthy to mention that LO cultures were not anaerobic but rather at a microaerophilic state. The gaseous O_2_ levels decreased from 9% (day 10) to 4% (day 14) in LO fungal cultures, with only a slight increase by H_2_O_2_ addition (10% on day 11) (Fig. 1D). More notable is the accumulation of CO_2_ (12–14%) in these cultures, indicating a switch to microaerophilic state and fungal fermentative metabolism [11].

**Table 1:** Functional annotations and over-representation analysis of shared DEGs

Functional category	GeneRatio SharedUp	GeneRatio Genome	Fold-Enrichment	*p*.adjust
DEGs upregulated in AE and LO cultures after H_2_O_2_ treatment				
Small secreted protein (SSP)	**24/113**	**174/12,133**	**14.81**	**< 0.001**
Short-chain dehydrogenase/reductase (SDR)	**8/113**	**155/12,133**	**5.54**	**< 0.001**
Aldo-keto reductase (AKR)	**7/113**	**56/12,133**	**13.42**	**< 0.001**
GNAT family acetyltransferase	**5/113**	**28/12,133**	**19.17**	**< 0.001**
Class I adenylate-forming enzyme family protein	**2/113**	**16/12,133**	**13.42**	**0.038**
Old yellow enzyme (OYE)-like NADPH reductase	**2/113**	**13/12,133**	**16.52**	**0.031**
Terpene cyclase family protein	**2/113**	**17/12,133**	**12.63**	**0.038**
Carbohydrate-active enzyme (CAZy)	5/113	340/12,133	1.58	0.251
Cytochrome P450 enzyme (CYP450)	4/113	123/12,133	3.49	0.087
DUF6533 domain protein	4/113	187/12,133	2.30	0.152
Transcription factors (TF)	4/113	230/12,133	1.87	0.209
Class I SAM-dependent methyltransferase	2/113	40/12,133	5.37	0.114
Major facilitator superfamily (MFS) transporter	2/113	190/12,133	1.13	0.531
NTPase family	2/113	34/12,133	6.32	0.110
Hypothetical protein with unknown function	19/113	4142/12,133		
DEGs downregulated in AE and LO cultures after H_2_O_2_ treatment				
Small secreted protein (SSP)	**17/162**	**174/12,133**	**7.32**	**< 0.001**
CAZy	**15/162**	**340/12,133**	**3.30**	**< 0.001**
Heat-shock protein (HSP)	**7/162**	**54/12,133**	**9.71**	**< 0.001**
MFS transporter	**7/162**	**190/12,133**	**2.76**	**0.045**
AKR	**4/162**	**56/12,133**	**5.35**	**0.027**
Medium-chain dehydrogenase/reductase (MDR)	**4/162**	**65/12,133**	**4.61**	**0.040**
Hydrophobin	**3/162**	**25/12,133**	**8.99**	**0.021**
Opsin family protein	**3/162**	**6/12,133**	**37.45**	**< 0.001**
TF	4/162	230/12,133	1.30	0.446
CYP450	2/162	123/12,133	1.22	0.570
Cytochrome b5-like protein	2/162	17/12,133	8.81	0.061
Hypothetical protein with unknown function	43/162	4142/12,133		

Functional categories with at least two differentially expressed genes under both atmospheric culture conditions after hydrogen peroxide treatment are shown, and functional categories with statistically significant enrichment (p.adjust < 0.05) based on the ORA are indicated in boldface. GeneRatio SharedUp, ratio of genes in the respective functional category to total SharedUp genes; GeneRatio Genome, ratio of total number of genes in the respective functional category in the genome to the total number of detected expressed genes in the genome; FoldEnrichment, enrichment of genes belonging to category in the SharedDEG set; p.adjust, p-value for the enrichment test adjusted with the Benjamini-Hochberg method

#### Shared upregulated genes

Among the 113 genes categorised as shared upregulated genes (Fig. 2), 24 genes were predicted to encode small secreted proteins (SSPs; [63]) whereas 19 genes code for hypothetical but unknown proteins (Table 1; Fig. 4). SSPs are defined here as < 250 aa proteins with an N-terminal secretion signal peptide and no recognized protein domains other than in some cases a CFEM domain, Nis1 domain, WSC domain or a LysM domain. Among these SSPs, six genes encode 68-aa proteins, and two putative SSPs (PRA_061090 and PRA_131600) shared no protein homology with entries in the ClusteredNR blast database with default search settings. Most of the SSP genes were strongly upregulated and included in the Top50 upregulated gene sets (Additional File 3). One such SSP gene (PRA_123220) was also significantly upregulated still on day 14 under both culture conditions, while another one (PRA_137980) remained differentially expressed only under AE conditions (Fig. 4).

There were 34 genes included in the Top50 upregulated gene sets both from AE and LO conditions (Additional File 3), including 13 SSP-encoding genes which, in turn, included all the aforementioned 68-aa SSPs. These 68-aa proteins share high similarity (75.3–97.5% identical at nucleotide (nt) level; 76.4–100% identical at amino-acid (aa) level). Interestingly, in addition to the hypothetical SSPs, one gene predicted to encode a secreted LysM effector as well as an adjacent CAZy GH18 chitinase-coding gene (Fig. 3) were both upregulated (with TPM counts rising from below 20 to more than 500). The Top50 upregulated genes also comprised genes encoding a UstYa family oxidase (gene PRA_096220), and one Old Yellow Enzyme (OYE) family oxidoreductase (gene PRA_059570). OYEs are participating in ROS resistance [64].

For DEGs with a predicted functional annotation, the most abundant protein classes represented after the SSPs were SDR (short-chain dehydrogenase/reductases), AKR (aldo/keto reductases), GNAT family acetyltransferases, CAZys, cytochrome P450 enzymes, DUF6533 domain proteins, and Transcription factors (Table 1). Among these, the SSPs, SDRs, AKRs, and GNAT family acetyltransferases were over-represented in the sharedUp gene set, as were also Class I adenylate-forming enzymes, OYE-like proteins and terpene cyclase family proteins. Worth mentioning is also that the transcription factors (TFs) were of three different types (C2H2-type, TEA and Zn(II)2Cys6 types) (Additional file 3).When the shared upregulated CAZy genes were inspected more closely, we found that auxiliary oxidoreductases were represented by classes AA3 (GMC superfamily oxidases; genes PRA_024090 and PRA_072760) and AA5 ) (PRA_118450), together with two genes (PRA_125140 and PRA_106400) predicted to encode a GH18 chitinase and a GH79 protein, respectively (Fig. 3). Among all the CAZy genes upregulated under any of the two conditions, genes coding for proteins of the auxiliary activity classes were more abundant compared to other CAZy classes.

Out of the 59 genes putatively encoding *P. radiata* AKRs, seven genes were upregulated under both AE and LO conditions (Table 1; Fig. 5) and showed a relatively high expression still on day 14 in the treated samples, four of them even as significantly differentially expressed under either one or both conditions on day 14. One of the predicted AKRs (PRA_108010) may be classified to the AKR7 subfamily (reduction of aldehydes, 7 predicted in the genome), two to the AKR13 subfamily (PRA_105620 and PRA_105730, identified through the KEGG annotation as pyridoxine 4-dehydrogenase/pyridoxal reductase [EC:1.1.1.65] genes, 7 predicted in the genome), two to the AKR9 subfamily (PRA_053820 and PRA_089060, 11 predicted in the genome, varying functions, including secondary metabolite production), one (PRA_043410) to the AKR8 subfamily (pyridoxal reductases, 4 predicted in the genome) and one (PRA_072220) to the AKR1-5 like subfamily (10 predicted in the genome). None of these annotated aldo/keto reductases possessed any secretion signal peptide, indicating that these enzymes are intracellularly located.

#### Shared downregulated genes

Among the 162 shared downregulated DEGs, 1/4 of the protein-coding genes (*n* = 43) could not be assigned any function (hypothetical proteins), whereas a set of 17 genes were predicted to encode SSPs (Table 1; Fig. 4). Among these there were five 88-aa SSPs, all containing a CFEM-like domain [65] with eight characteristically spaced cysteine residues. These genes shared a high degree of similarity (86.5–97.7% nt sequence identity; 87.5–97.7% aa sequence identity) and a few of them were located close to each other in the genome (genes PRA_106940, PRA_106960, PRA_107020). These findings point to co-regulation and recent gene duplication events. Four of the shared downregulated 88-aa SSPs were also shared in the sets of Top50 downregulated genes (Additional File 3), including one gene (PRA_106960) that remained significantly downregulated still on day 14 under AE conditions (Fig. 4). In total, 32 out of the 162 shared downregulated DEGs were shared between the Top50 downregulated genes under both AE and LO culture conditions.

The shared downregulated DEGs included a total of 15 genes encoding CAZymes (carbohydrate active enzymes) (Table 1; Fig. 3). Most of these represented GH glycoside hydrolases of several classes (GH 3, 15, 16, 25, 28, 43, 152). Other CAZy classes were represented by one GT8 class glycosyl transferase (glycogenin GT8-type protein; gene PRA_059030, 1 out of 6 genes in the genome), one CE4 carbohydrate esterase (PRA_043870), one AA1_2 multicopper oxidase (PRA_004790) and three AA3 GMC oxidoreductases (genes PRA_046740, PRA_069620, PRA_046660) (Fig. 3). The GH25 class gene (PRA_101800) was significantly downregulated still on day 14 under AE conditions, whereas the AA1_2 class gene (PRA_004790) was significantly downregulated still on day 14 under LO conditions.

Well-represented among the shared downregulated DEGs were seven genes encoding heat shock proteins (HSPs), out of which five putative proteins could be classified as Hsp20 (Table 1, Additional File 3). Four of these were also found in the set of Top50 downregulated DEGs for both AE and LO conditions 18 h after the oxidative shock (Additional File 3). The HSP genes constituted one out of four functional categories containing more than three DEGs where all included genes were downregulated (Fig. 5). Another group of seven genes in the shared downregulated set of DEGs encode putative major facilitator superfamily (MFS) transporters (Table 1; Fig. 5), among which were four predicted to encode hexose MFS proteins (4/25 annotated genes with a cd17356 domain). There were four transcription factor genes present among the sharedDown genes, all representing different types of TFs (Zn(II)2Cys6, RING finger, Opi1 and BHLH types). In addition, there was one shared downregulated gene (PRA_066950) encoding a stress responsive protein (Additional File 3). Medium-chain dehydrogenase/reductase (MDR) superfamily proteins were represented by four shared downregulated genes (Table 1; Fig. 5). Two of these were annotated as genes of the CAD3 family (related to cinnamyl alcohol dehydrogenases), represented by three genes annotated in the *P. radiata* genome. The third gene was significantly downregulated after oxidative treatment under AE conditions only. Downregulation of the MDR-encoding genes points to suppression of fungal aromatic metabolism (interconversion of aromatic alcohols and aldehydes or ketones) by the oxidative treatment. This effect could not be seen in the biosynthetic production of veratryl alcohol, which continued to accumulate in AE and LO cultures irrespective of H_2_O_2_ addition (Fig. 1G). However, the concentration of veratraldehyde in the culture supernatant increased on day 11 and returned to day 10 levels by day 14 (Fig. 1H).

### Atmosphere-dependent DEGs responsive to oxidative treatment

Among the DEGs that were significantly upregulated only in fungal AE cultures after H_2_O_2_ treatment (day 11 vs. day 10; 153 genes) we found 12 genes coding for SSPs (Fig. 4), out of which 3 genes were included in the Top50 most drastically upregulated genes under these conditions (Additional File 3). The Top50 upregulated genes also included 3 genes encoding oxidoreductases and one gene encoding an alpha/beta hydrolase that were differentially expressed in AE fungal cultures only. Among the functional categories with > 3 DEGs, the WD40 repeat domain-containing proteins stood out as a category with upregulated genes only, and under AE conditions specifically (Fig. 5). Moreover, among the DEGs included in the SDR category, 5 genes were significantly upregulated under AE conditions only, and 4 of these were upregulated still on day 14 (Fig. 5).

The genes downregulated under AE conditions uniquely also included a few functionally interesting genes (Additional File 3). There were genes predicted to encode a general stress protein of the pyridoxamine 5’-phosphate oxidase (PPOX) class (gene PRA_014810), malate synthase (PRA_014060), isocitrate lyase (PRA_086140), phosphoenolpyruvate carboxykinase (PRA_020410), putative CAZy GH16 galactosidase (PRA_062250), and a predicted secreted S1/P1 nuclease. In this gene (PRA_117980), however, the S1/P1 nuclease domain is disrupted by RiPP-like (ribosomally synthesized and post-translationally modified peptide-like) repeats.

In the Top50 upregulated gene set expressed under LO conditions, there were six genes significantly upregulated only under these conditions (Additional File 3). Among them was one gene (PRA_100950) encoding a hemerythrin domain-containing protein (class signal transduction and oxygen-carrier hemerythrins) [66] and a gene encoding a putative Global transcription regulator sge1 (PRA_073620). The latter was the only significantly upregulated Gti1/Pac2 gene of *P. radiata*. Gti1/Pac2 proteins are involved in responses to H_2_O_2_, in production of phytotoxins or secondary metabolites, biosynthesis of trichothecene toxins and regulation of expression of SIX (*S*ecreted *I*n *X*ylem) effector genes [67].

Genes that were significantly downregulated and found among the Top50 downregulated gene set under LO conditions only included genes encoding a Ricin B lectin domain-containing protein (a carbohydrate binding protein; PRA_107410), an RTA1-domain-containing protein (a transmembrane transport protein; PRA_026610), one hydrophobin (PRA_012160) and CAZy GH5 enzyme (PRA_024450) (Additional File 3). Overall, there were substantially more CAZy genes downregulated under LO conditions compared to AE conditions on day 11 (Fig. 3). Noteworthy is also that all the DEGs annotated as Ricin domain-containing proteins were significantly downregulated under LO conditions only (Fig. 5).

### Downregulation of carbon metabolism genes

**Fig. 7:**
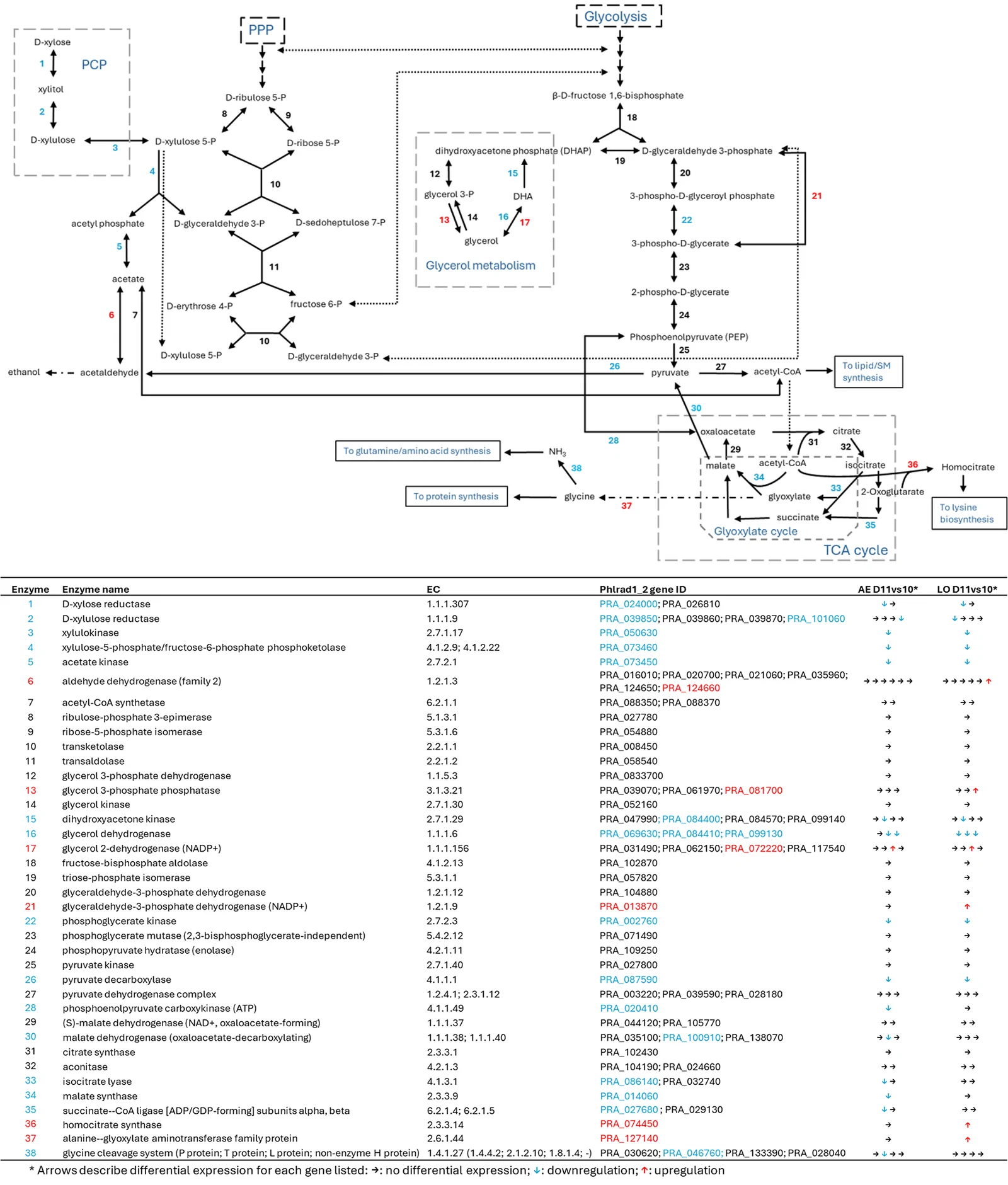
Effect of H_2_O_2_ treatment on core carbon metabolic pathways in *Phlebia radiata* birch wood substrate cultures. Enzymes involved in pentose phosphate pathway (PPP), pentose catabolic pathway (PCP) and glycolysis in connection to glycerol metabolism and downstream processes are depicted with numbers explained in the lower table with respective EC numbers and annotated genes in the *P. radiata* genome (genes PRA_*). Only statistically significant up- or downregulation according to our DE analysis is emphasized with colouring. DHA, dihydroxyacetone; SM, secondary metabolite; TCA, tricarboxylic acid (Kreb’s) cycle. D11vs10, difference in gene expression before (day 10) and after (day 11) exposure to H_2_O_2_

Genes involved in carbon metabolism were also represented among the shared downregulated genes, including genes encoding enzymes involved in pentose catabolic pathway (PCP) and glycerol metabolism (Fig. 7). These also include a phosphoglycerate kinase (involved in glycolysis), a pyruvate decarboxylase catalysing the irreversible, non-oxidative decarboxylation of pyruvate into acetaldehyde (on route to production of ethanol), and an acetate kinase (operating in acetyl-CoA precursor synthesis). Some of these carbon metabolism-related genes were also included in the Top50 sets of downregulated genes after oxidative treatment under both AE and LO culture conditions (Additional File 3).

In addition, genes linked to pentose and glucuronate interconversions were detected among the shared downregulated genes. Among these were genes encoding a xylulokinase (PRA_050630; one of the two putative genes) and a 2-dehydro-3-deoxy-D-gluconate 5-dehydrogenase, a Ga5DH-like classical short-chain dehydrogenase/reductase (PRA_101040). Expression of the only gene encoding xylulose 5-phosphate phosphoketolase (Xfp; PRA_073460) in *P. radiata* was also significantly downregulated after oxidative treatment. Together with Xfp, among the downregulated DEGs in *P. radiata* were two genes (under AE conditions) to all three genes (under LO conditions) encoding glycerol dehydrogenases, which together with one downregulated dihydroxyacetone (DHA) kinase further indicate changes in glycerol production (Fig. 7). A glycogen phosphorylase, involved in breakdown and utilization of the fungal glucose storage molecule glycogen, was also downregulated after the treatment. This furthermore indicates decline in utilization of alternate or storage carbon sources, together with suppression of pentose catabolism.

## Discussion

Investigations of fungal response to oxidative stress at molecular and genetic level have particularly focussed on human and plant pathogens including species of Ascomycota (*Candida spp.*,* Aspergillus spp.*,* Fusarium oxysporum*,* Talaromyces marneffei*,* Magnaporthe grisea*), a few Basidiomycota (*Cryptococcus spp.*,* Ustilago spp.*, rust fungi), and on the non-pathogenic model yeast *Saccharomyces cerevisiae* [1, 64, 68]. For the cellular sensor pathways, protective and adaptive responses are activated by various stress conditions, and heat shock proteins (HSPs) are specifically involved [69]. Gene knock-out experiments have demonstrated, however, differences in the functional effectiveness and specificity of response and transcription factor regulators among fungal species.

Saprotrophic fungi, including the wood decay Basidiomycota, are yet underexplored regarding their genetic response and metabolic adaptability to changes in abiotic effects like oxidative stress. Previously, the white rot fungus *Phlebia radiata* was subjected to oxidative treatment on birch wood substrate under two experimental conditions (aerobic versus oxygen-limited) to observe the effect of oxidative shock on fungal metabolism and activities [17]. In the current study, we report on changes in fungal gene expression and specific metabolic pathways caused by oxidative stress under similar conditions. Birch wood as substrate simulates natural-like growth habitat and nutritional conditions for the fungus [33, 70] thereby allowing expression of fundamental enzymes operating in essential cellular processes.

During wood decomposition, extracellular ROS (reactive oxygen species) such as hydrogen peroxide is generated and exploited by the fungi [11, 14, 71]. Fungal redox enzymes such as the flavin-containing CAZy auxiliary activity oxidases in the GMC protein family provide hydrogen peroxide to support oxidative chemistry and catalysis of other extracellular redox enzymes, such as Class-II peroxidases and lytic polysaccharide monooxygenases, especially utilized by white rot fungi in lignocellulose decomposition [2, 14, 30]. Hydrogen peroxide has been recognised as a defensive or signalling molecule in fungal interspecies interactions [72, 73] and as an enhancer of production of fungal secondary metabolites [74], thereby confirming the universal regulative role of H_2_O_2_ in intracellular metabolism and cellular processes. However, exogenous hydrogen peroxide may even cause deleterious effects on fungal secreted enzymes by activity inhibition or protein post-translational modification, leading to loss of function [13, 18]. This may explain the sharp decline of laccase and manganese-peroxidase activities observed in our study one day after the oxidative treatment. Continuous production of the secondary metabolite veratryl alcohol, on the other hand, is evidencing fungal vitality and tolerance against the oxidative shock. In this respect, it may be assumed that wood decay fungi possess effective and versatile cellular and genetic mechanisms for mitigation of ROS and oxidative stress.

Notable in metabolic responses of *P. radiata* to the hydrogen peroxide-generated oxidative shock in our study was suppression of key enzymes involved in pentose metabolism. Under both atmospheric conditions, downregulation at gene expression level of xylose reductase and xylulokinase enzymes was observed, in addition to downregulation of one xylulose reductase, thereby indicating a decline in pentose metabolism (Fig. 7). Xylulokinase catalyses the rate-limiting step in ATP-dependent phosphorylation of D-xylulose to D-xylulose 5-phosphate, which is associated with regulation of carbohydrate metabolism and genes involved in pentose catabolic (PCP) and pentose phosphate (PPP) pathways in the Ascomycota species *Aspergillus niger* [75]. Downregulation of xylulokinase in *A. niger* is connected to carbon catabolite repression with excess glucose, albeit not directly mediated by the well-known carbon catabolite repressor CreA but involving other regulators [76]. Furthermore, expression of the only gene encoding xylulose 5-phosphate phosphoketolase (Xfp) in *P. radiata* was significantly downregulated after oxidative treatment. Xfp is (one of the enzymes) responsible for linking PPP to glycolysis and glycerol metabolism, by producing acetyl phosphate and glyceraldehyde-3-phosphate from xylulose-5-phosphate (Fig. 7). The key role of Xfp in fungal carbohydrate metabolism may extend to pathway control by enzyme allosteric regulation and substrate cooperativity as has been found in the pathogenic basidiomycete *Cryptococcus neoformans* [77].

Differences in *P. radiata* gene expression patterns after H_2_O_2_ treatment between aerobic and low oxygen culture conditions noticed in this study may be explained by dissimilarity in the fungal metabolic state before experiencing the oxidative shock, as was observed in enzyme and biochemical activities, and headspace gas content in the fungal low-oxygen cultures. Concerning carbon catabolic metabolism, several genes encoding MFS hexose transporters (4 out of 25 genes annotated in the genome) were downregulated as a response to oxidative treatment pointing to reduced intake of sugars. It is noteworthy to mention that on solid lignocellulose substrates, the fungus is efficient in enzymatic degradation of cellulose and hemicelluloses leading to accumulation of sugars in the culture supernatant [11, 17, 26, 30]. Downregulation of sugar transporters, pentose catabolic pathway and glycerol metabolism (Fig. 7) supports the interpretation that the oxidative treatment released wood lignocellulose compounds providing an extra burst of hexoses and saccharides available for the fungus, as was found in our previous study under similar conditions on birch-wood substrate [17].

Strikingly, we observed that several genes related to stress response such as chaperones and genes related to DNA repair were downregulated as a response to oxidative shock. Among these were five genes encoding Hsp20 proteins (Hsp20 family, small heat shock proteins sHSPs) and a protein chaperone of ClpB/HSP104 subfamily. Hsp20 sHSPs are diverse in function, 12 to 42 kDa in size, and abundant in all organisms [78]. Hsp104 in turn are important anti-stress proteins in *S. cerevisiae* especially after heat shock, functioning as molecular chaperones guiding protein folding but triggering protein aggregation at specific conditions [79]. The unexpected downregulation of several chaperones in *P. radiata*, however, may indicate that oxidative treatment inflicted less severe intracellular damage requiring activation of protein repair mechanisms.

This notion in combination with the finding that specific redox enzyme-encoding genes were upregulated in *P. radiata* by the treatment may indicate that oxidative shock induces a more unique response in the fungus compared to responses to other stress factors, such as heat. Downregulation of Hsp104 may be an assurance to sustain proteins and enzymes proper in structure to allow essential cellular functions (and repair mechanisms) to take over directly after the shock. Among the genes downregulated by oxidative treatment under both atmospheric conditions were also genes predicted to encode a heat shock factor family protein (PRA_047660) and a hybrid histidine kinase/response regulator (PRA_047620). Therefore, their involvement in oxidative-stress related regulation of the differentially expressed heat shock proteins could be postulated.

Treatment with H_2_O_2_ resulted in upregulation of several genes in *P. radiata* encoding intracellular redox-enzymes with annotated functions such as aldo/keto reductases and retinol dehydrogenase-like short chain dehydrogenase/reductases. Among these were many enzymes specific for antioxidant molecules (e.g. ubiquinone and retinol) and participating in antioxidation reactions in lipid membranes [80, 81], therefore possibly reflecting remediating reactions after lipid peroxidation and damage caused by hydrogen peroxide and ROS. An array of highly overexpressed genes belonging to short chain dehydrogenases and aldo/keto reductases was similarly upregulated in another Basidiomycota Polyporales species, *Pycnoporus coccineus*, when subjected to interactions with other fungal species [82]. These findings may strengthen the idea of oxidative stress triggering responses similar to those observed in fungal interspecies interactions [72, 73].

Aldo/keto reductases form a protein superfamily presenting a wide substrate range, with e.g. involvement in detoxification of reactive aldehydes and ketones to less reactive alcohols, and in fungal carbon metabolism [83, 84]. In the fungal cultures with wood as substrate, substrate-derived compounds may accumulate and become oxidized and/or modified by hydrogen peroxide treatment, as was observed previously [17]. The considerably lower iron reduction capacity of the fungal culture supernatants in comparison to the no-fungus wood-substrate control incubations, and the significant loss of this activity following the oxidative treatment, are well in line with observations of decreasing numbers of aromatic compound peaks in the supernatant. Furthermore, many of the wood substrate-derived compounds possess bioactive and ROS-quenching antioxidant properties [85].

Several genes encoding SSPs (small secreted proteins), especially a few for putative 68-aa SSPs, were among the most significantly upregulated DEGs after the oxidative treatment whereas a few other SSP-encoding genes, like the 88-aa proteins containing a CFEM domain [86], were conversely downregulated. For many of the upregulated SSPs, their expression returned to baseline within three days after the treatment. This indicates quick stress-responsive functions for the specific *P. radiata* SSPs. Since SSPs are numerous in fungal secretomes with genes universally recognised in genomes across fungal taxa, they are involved in various physiological and interactive processes. Together with their known roles as effector proteins in pathogen-host interactions, SSPs are involved in nutritional cycles and in modulating microbiomes and fungal growth [63, 87]. These findings imply that especially the significantly upregulated SSPs may be involved in quick metabolic adaptation and mitigation of ROS and oxidative shock in wood-decay fungi of Basidiomycota.

Under aerobic conditions, one *P. radiata* gene encoding a potential SSP of LysM effector type (gene PRA_063310) was significantly upregulated after exposure to hydrogen peroxide. LysM (Lysin motif) effectors are carbohydrate binding module (CBM) proteins with essential roles in fungi [88]. In plant pathogenic fungi, LysM effectors confer protection of fungal hyphae by binding to cell wall chitin, thereby preventing host plant’s detection and degradation by chitinases [89, 90]. Similar suppression of host defence by LysM effector proteins may increase virulence of mammal-pathogenic fungi [91]. In this context, the simultaneous upregulation of genes encoding a chitinase enzyme (gene PRA_125140) and LysM effector in *P. radiata* as a response to oxidative shock is interesting; it may be suggested that the fungus expresses stress-induced chitinase against other fungi together with an effector protein which protects its own hyphae against chitinase attack. This observation is another example of various genetic and cellular regulatory mechanisms of the fungus to quickly react against oxidative shock and stress.

## Conclusion

Our study indicates that instead of an overall dramatic transcriptomic silencing with broad downregulation of gene expression and essential cellular processes, the fungus responds to the oxidative stress more specifically by upregulation of genes encoding small secreted proteins, aldo-keto reductases, and a few hydrolytic CAZymes, together with downregulation of carbon catabolic and branching metabolic pathways, especially involved in pentose and glycerol metabolism (Fig. 7). Changes in culture atmosphere and oxygen content apparently influenced metabolic pathways related to carbon usage and cofactor regeneration. These observations pinpoint simultaneous regulation based on the substrate (available carbon sources) and transient abiotic factor (oxidative stress), operating to quickly adapt and recover from the oxidative shock effect. Our experiments furthermore highlight adaptation of the fungus on wood as its habitat and substrate, which is sustaining existence and metabolism even under stress and changing atmospheric conditions.

## Supplementary Information

Supplementary Material 1. Table of RNA-Seq raw counts.

Supplementary Material 2. PCA of RNA-Seq read counts.

Supplementary Material 3. Tables of DEG analysis results including gene functional annotation.

## Data Availability

Data generated or analysed during this study are included in the published manuscript or Additional Files. Raw RNA-Seq data are stored in the European Nucleotide Archive ENA at EMBL-EBI under accession PRJEB89221. Updated Phlebia radiata 79 (FBCC 0043) genome has been deposited in ENA EMBL-EBI under accession PRJEA74265 (assembly GCA_979071105).
